# Possible Genetic Determinants of Response to Phenytoin in a Group of Colombian Patients With Epilepsy

**DOI:** 10.3389/fphar.2020.00555

**Published:** 2020-05-07

**Authors:** Carlos Alberto Calderon-Ospina, Jubby Marcela Galvez, Claudia López-Cabra, Natalia Morales, Carlos Martín Restrepo, Jesús Rodríguez, Fabio Ancízar Aristizábal-Gutiérrez, Alberto Velez-van-Meerbeke, Paul Laissue, Dora Janeth Fonseca-Mendoza

**Affiliations:** ^1^ Center for Research in Genetics and Genomics (CIGGUR), GENIUROS Research Group, School of Medicine and Health Sciences, Universidad del Rosario, Bogotá, Colombia; ^2^ Department of Pharmacy, Faculty of Sciences, Universidad Nacional de Colombia, Bogotá, Colombia; ^3^ Neurology Department, Hospital Universitario Mayor Mederi, Bogotá, Colombia; ^4^ Neuroscience Research Group (NEUROS), School of Medicine and Health Sciences, Universidad del Rosario, Bogotá, Colombia; ^5^ Cardioinfantil Foundation, Institute of Cardiology, Bogotá, Colombia; ^6^ Department of Development, Reproduction and Cancer, INSERM U1016 Institut Cochin, Paris, France; ^7^ Biopas Laboratoires, BIOPAS GROUP, Bogotá, Colombia

**Keywords:** drug resistant epilepsy, phenytoin, drug-related side effects and adverse reactions, pharmacovigilance, pharmacogenetics

## Abstract

**Background:**

Epilepsy is a serious health problem worldwide. Despite the introduction of new antiepileptic drugs (AEDs) almost 30% of these patients have drug-resistant forms of the disease (DRE), with a significant increase in morbi-mortality.

**Objective:**

Our objective was to assess the impact of some genetic factors and its possible association with treatment response and adverse drug reactions (ADRs) to phenytoin in 67 adult Colombian patients with epilepsy.

**Methods:**

We conducted an analytical, observational, prospective cohort study to screen four polymorphisms in pharmacogenes: CYP2C9*2-c.430C>T (rs1799853), CYP2C9*3-c.1075A>C (rs1057910), *ABCB1-c.3435T>C* (rs1045642), and *SCN1A-IVS5-91G>A* (rs3812718), and their association with treatment response. Patients were followed for 1 year to confirm the existence of DRE (non-response) and ADRs using an active pharmacovigilance approach, followed by a consensus in order to classify ADRs according to causality, preventability, intensity and their relation with phenytoin dose, the duration of treatment, and susceptibility factors (DoTS methodology).

**Results:**

A little more than half of evaluated subjects (52.2%) were non-responding to phenytoin. Regarding the genotype-phenotype correlation there was no association between polymorphisms of *SCN1A* and *ABCB1* and DRE (non-response) (*p* = 0.34), and neither with *CYP2C9* polymorphisms and the occurrence of ADRs (*p* = 0.42). We only found an association between polymorphic alleles of *CYP2C9* and vestibular-cerebellar ADRs (dizziness, ataxia, diplopia, and dysarthria) (*p* = 0.001). Alleles CYP2C9*2-c.430C>T and CYP2C9*3-c.1075A>C were identified as susceptibility factors to ADRs in 24% of patients.

**Conclusions:**

Decreased function alleles of *CYP2C9* were highly predictive of vestibular-cerebellar ADRs to phenytoin in our study (*p* = 0.001). However, the genetic variants CYP2C9*2-c.430C>T, CYP2C9*3-c.1075A>C, *ABCB1-c.3435T>C,* and *SCN1A-IVS5-91G>A*, were not associated with treatment response in our study.

## Introduction

Epilepsy is one of the most common chronic neurological disorders (Thurman et al., 2011). It affects around 65 million people around the world, and approximately 80% of these patients live in developing countries (Thurman et al., 2011; Ngugi et al., 2011). According to the World Health Organization (WHO), epilepsy represents 0.75% of the global burden of disease, particularly in children and young adults, with an estimated 23.4 million cases of active epilepsy in 2015 (Feigin et al., 2017). The most serious form of the disease, which is refractory to pharmacological treatment, represents approximately 30% of the cases (Cavalleri et al., 2011; Balestrini and Sisodiya, 2018).

Despite the introduction of new antiepileptic drugs (AEDs), more than 30% of patients with epilepsy have drug-resistant forms of the disease, which leads to a significant increase in the morbidity and mortality of epilepsy (Thurman et al., 2011).

This form of the disease, known as drug-resistant epilepsy (DRE), has been defined by the *International League Against Epilepsy* (ILAE) as “failure of adequate trials of two tolerated and appropriately chosen AED schedules (whether as monotherapies or in combination) to achieve seizure freedom” (Kwan et al., 2009). Although nowadays there are more than 30 different AEDs, there is no satisfactory treatment for DRE (Cavalleri et al., 2011). Therefore, patients with epilepsy tend to be difficult to treat due to the need for combination therapy and the high frequency of non-responding patients, drug interactions, and adverse drug reactions (ADRs), among other factors (Manford, 2017).

On the other hand, genetic factors have been involved as possible causes of variable responses to AEDs, including a lack of response to treatment, as well as the occurrence of ADRs (Balestrini and Sisodiya, 2018). This genotype-phenotype relationship is particularly well studied for phenytoin, which is one of the most widely used AEDs in the world (Cavalleri et al., 2011; Caudle et al., 2014; Balestrini and Sisodiya, 2018). However, the relationship between the therapeutic response and the most widely studied genetic variants, is mainly unknown (Balestrini and Sisodiya, 2018). It is the same case for some ADRs to phenytoin (e.g., cutaneous ADRs) (Balestrini and Sisodiya, 2018).

Likewise, among the most important pharmacogenes which influence the response to phenytoin are those related to the transport through the blood–brain barrier, such as the *ABCB1* gene, which encodes the glycoprotein P (P-gp); the *CYP2C9* gene and others that encode drug-metabolizing enzymes, and the genes that encode targets, such as *SCN1A,* which encodes the α-subunit of the voltage-gated sodium channel, which is a common target for phenytoin and other AEDs (McDonagh et al., 2011; Balestrini and Sisodiya, 2018).

In the new era of personalized medicine, it is essential to identify possible pharmacogenomic biomarkers as predictors of treatment response (El-Ibiary et al., 2008). Therefore, we evaluated the impact of the best-studied variants of the aforementioned genes as possible risk factors for non-response or ADRs to phenytoin.

### Aim of the Study

The aim of the study was to assess the impact of genetic factors as causes of non-response and ADRs to phenytoin in a group of Colombian patients with epilepsy.

## Materials and Methods

### Study Design

This was a cohort observational study that followed patients for 12 months, conducted jointly by treating physicians, and members of the study team, which was composed of a pharmacist, a pharmacologist, and doctors of other specialities (genetics, epidemiology, neurology).

### Setting and Participants

Screening was carried out on patients who were treated at a center specializing in the treatment of patients with epilepsy and at the neurological service of a tertiary-level university hospital in Bogotá between October 2012 and February 2014.

Regarding the sample size calculation, we estimated a prevalence of 0.3% of DRE in Colombia according to what is reported in the literature (Pradilla et al., 2003). The value of sample proportion (p) was estimated according to the frequency of *ABCB1-c.3435T>C (rs1045642)* allele identified in gnomAD database (https://gnomad.broadinstitute.org/). Considering an alpha value of 0.05 and a power of 90% the sample size was equal to 68.

Patients with an established diagnosis of epilepsy, resident in Colombia, who had given written consent for their participation, and who were using at least one AED were included in the study. The patients were monitored for 12 months, with the aim of establishing the phenotypic response to treatment. Subjects who met the ILAE criteria were considered as non-responding patients (DRE) (Kwan et al., 2009), while those that did not suffer any epileptic seizure during the treatment period were deemed to be responsive (Kwan and Brodie, 2000). Patients with impaired hepatic and/or renal function, as well as those who did not adhere to the treatment, were excluded from the study. Ninety-six patients were included in the study. However, only 77 patients were evaluated since 19 of them were lost during the follow-up, so the phenotype could not be adequately assessed. Of these 77 patients, ten more were excluded because they were not receiving phenytoin, for a final sample size of 67 patients. The study protocol was approved by the Ethics Committees of the participating institutions and was carried out in accordance with the Helsinki Declaration, in addition to local and international regulations regarding research on human subjects.

### Research Procedure

#### *ABCB1*, *SCN1A*, and *CYP2C9* Genotyping

Genomic DNA was obtained from patient’s peripheral blood samples using the standard salting-out procedure. Genomic regions encompassing CYP2C9*2-c.430C>T (rs1799853), CYP2C9*3-c.1075A>C (rs1057910), *ABCB1-c.3435T>C* (rs1045642), and *SCN1A-IVS5-91G>A* (rs3812718) polymorphisms were amplified by PCR. Specific primers for PCR reactions were designed using Primer Blastn (https://www.ncbi.nlm.nih.gov/tools/primer-blast/) and Primer3 software (https://primer3plus.com/cgi-bin/dev/primer3plus.cgi). For sequencing reactions we used an internal primer designed by Primer3 software. Primer sequences are available upon request. For each PCR reaction, 200 ng of DNA was using, 12.5 µl of the GoTaq Green Master Mix (Promega M-712) and 2 µl of each primers (forward and reverse at 10 µM) were added to each sample. Thereafter PCR was performed and consisted of initial denaturation for 10 min at 95°C, then 35 amplification cycles, (95°C for 40 s, 60°C (for ABCB1, SCN1A, and CYP2C9 *3), or 55°C (for CYP2C9*2) for 40 s and 72°C for 1 min), followed by a 10-min incubation at 72°C. The amplified products were visualized on ethidium bromide stained agarose gels.

PCR products were purified with conventional procedures (using shrimp alkaline phosphatase and exonuclease I). Direct Sequencing was performed with the same primers used in PCR reaction. Sequencing was made using the 3130xl Genetic Analyzer (Applied Biosystems). We analyzed the sequences in ab1.file by using FinchTV software (http://www.digitalworldbiology.com/finchtv). Wild-type sequences are listed in the Ensembl database (https://finchtv.software.informer.com): ENST00000260682.7 (*CYP2C9*), ENST00000622132.4 (*ABCB1*), ENSG00000144285 *(SCN1A*). Alignments of sequences were realized by using of ClustalW software.

### Outcome Measures

Patients were prospectively evaluated to establish the phenotypic response to treatment. This evaluation was carried out by means of a monthly telephone call and the completion of a monitoring form for each patient included in the study. As an additional endpoint ADRs were assessed by a checklist (**Supplementary Data Sheet 1**) designed for the study and applied by a pharmacologist with extensive experience in pharmacovigilance, who systematically checked for possible ADRs in each telephone call. Each time that an ADR was suspected, we obtained additional information from the patient’s medical records and data provided by the treating physician.

Each ADR was subsequently evaluated in terms of its causality using the Naranjo algorithm (Naranjo et al., 1981), preventability by the Schumock and Thornton criteria (Schumock and Thornton, 1992), the Aronson and Ferner intensity criteria (Aronson and Ferner, 2005), and the DoTS (dose, time, and susceptibility) criteria from the same authors (Aronson and Ferner, 2003).

The Naranjo algorithm is a questionnaire proposed by Naranjo et al. for determining the likelihood of whether an ADR was caused by the suspect drug (phenytoin in our study) rather than concomitant factors (e.g., comorbidities). Probability was assigned using a score named definite, probable, possible, or doubtful. Whenever the answer to any of the Naranjo algorithm questions was unknown, a score of “0” was assigned to the corresponding question, as it’s established in the algorithm (Naranjo et al., 1981). Schumock and Thornton criteria are a set of seven questions widely used in pharmacovigilance studies which are useful in determining the preventability of ADRs. Anytime that the answer to one or more of the seven questions was “yes” the ADR was classified as preventable (Schumock and Thornton, 1992). The intensity criteria of Aronson and Ferner evaluate the severity of ADRs based on the need to change the dosage regimen of the offending drug (phenytoin in our study): grade 1 (no change in dosage regimen was required), grade 2 (altered dosage regimen was required or desirable), and grade 3 (withdrawal was required or desirable) (Aronson and Ferner, 2005). Finally, the DoTS criteria are a classification system for ADRs based on dose responsiveness, time course, and susceptibility (Aronson and Ferner, 2003). Regarding the dose, ADRs may have occurred at supratherapeutic doses (toxic effects); standard therapeutic doses (collateral effects); or subtherapeutic doses in susceptible patients (hypersusceptibility reactions). We defined standard therapeutic doses in our study as the defined daily dose (DDD) of phenytoin set by the WHO which is 300 mg. In relation to time, ADRs can be time-independent which occurred at any time during treatment, independent of the duration of therapy, or time-dependent ADRs. In turn, time dependent ADRs were classified as rapid, first dose reactions, early, intermediate, late, or delayed depending on their temporal relationship with the administration of phenytoin. At last, the DoTS system include six susceptibility factors that could increase the risk of ADRs in certain populations (e.g., malnourished elderly patients). In our study these factors were: age (patients 60 years or older), sex (female gender), genetic variation (CYP2C9*2-c.430C>T and CYP2C9*3-c.1075A>C genetic polymorphisms), exogenous factors (drug interactions), and disease (comorbidities that, according to the researchers’ criteria, could predispose to ADR) (Aronson and Ferner, 2003). Genetic susceptibility for ADRs was carried out considering the presence of decreased function alleles in *CYP2C9* that confer the intermediate/poor metabolizer phenotype, and its association with certain ADRs that have been associated with supra-therapeutic concentrations of phenytoin (Cavalleri et al., 2011). Physiological factors (e.g., pregnancy), which is defined by Aronson and Ferner as an additional susceptibility factor, were not assessed because pregnant women were not included in our study.

Each case of ADR or non-response was detected by a pharmacologist and subsequently double-checked by a pharmacist. Two independent teams consisting of an epidemiologist, a clinical neurologist and a geneticist assessed the cases separately, and any discrepancies were resolved by consensus with a third author (pharmacologist).

### Statistical Analysis

The categorical variables were presented as frequencies with percentages, using 95% confidence intervals (CI 95%).

The allelic and genotypic frequencies for CYP2C9*2-c.430C>T, CYP2C9*3-c.1075A>C, ABCB1-c.3435T>C, and *SCN1A-IVS5-91G>A* were determined using the SNP-Stats software (https://www.snpstats.net/). Hardy-Weinberg equilibrium (HWE) was evaluated using chi-square test. We correlated *ABCB1* and *SCN1A* genotypes with the treatment response (phenotype) and the *CYP2C9* genotypes with ADRs (phenotype) using SPSS V25. Pre-specified analyses were performed for the different genotypes and ADRs by organs and systems affected. The result was deemed statistically significant when *p*<0.017 (Bonferroni correction).

## Results

**Table 1** presents the general characteristics of the 67 patients included, who had a mean age of 60 years. Approximately 51% of the patients were men, the majority of whom came from the Andean region of Colombia. In around 50% of cases, the epilepsy had an unknown cause and 90% of patients had no family history of epilepsy. Fifty-two percent of the subjects were classified as patients with DRE (non-responding patients). Sixty-four percent of the patients received antiepileptic treatment as monotherapy with phenytoin.

**Table 1:** Description of the population evaluated (n = 67).

Variable	n	%	CI 95%
Sex			
Female	33	49.3	37.7–60.9
Male	34	50.7	39.1–62.4
Age (years)			
18–64	35	52.2	40.5–63.8
65–79	22	32.8	22.8–44.8
≥80	10	14.9	8.3–25.4
BMI (kg/m^2^)			
< 18.5	8	11.9	6.2–21.8
18.5–24.9	22	32.8	22.8–44.8
25–29.9	33	49.3	37.7–60.9
≥30	4	6.0	2.4–14.4
Etiology of epilepsy			
Structural	31	46.2	3.5–58.1
Genetic	3	4.5	1.6–12.4
Unknown	33	49.3	37.7–60.9
Family history of epilepsy			
Yes	7	10.5	5.2–20.0
No	60	89.6	80.0–94.8
Response to antiepileptic treatment			
DRE (non-responding patients)	35	52.2	40.5–63.8
Controlled epilepsy	32	47.8	36.3–59.5
Drug treatment			
Monotherapy	43	64.2	52.2–74.6
Combination therapy	24	35.8	25.4–47.8

CI 95%, confidence interval 95%; BMI, body mass index; DRE, drug-resistant epilepsy.

### Prevalence of the Studied Genotypes

**Table 2** presents the allelic and genotypic frequencies of the polymorphisms analyzed.

**Table 2:** Allelic and genotypic frequencies of the studied genotypes.

Gene	Allelic frequency (%)		Genotypic frequency (%)	
*CYP2C9*	*1	86.4	*1/*1	74
	*2	9.1	*1/*2	18.2
	*3	4.5	*1/*3	6.5
			*3/*3	1.3
*ABCB1*	A	60	AA	36.4
	G	40	GA	44.2
			GG	19.5
*SCN1A*	C	39	CC	16.9
	T	61	TC	44.2
			TT	39

### Pharmacovigilance Analysis of the Studied Patients

We identified 53 patients with ADRs. Considering that some patients had more than one ADR the total number of ADRs was 143.

Regarding the systems/organs affected by ADRs, the nervous system was most affected, with 50.3% of the cases, followed by vestibular-cerebellar ADRs (18.2%) and gastrointestinal system (11.9%). The skin and the respiratory system were affected in 5.6% of ADR cases each of them. Reactions involving the endocrine/metabolic, hematological, genitourinary systems, and constitutional symptoms make up the remaining cases (8.4%).

**Table 3** presents the ADRs to phenytoin in the study patients.

**Table 3:** Description of adverse reactions to phenytoin (n = 143).

ADR	Number of cases	Percentage of total % (CI 95%)
**Nervous system**		
Somnolence	22	15.4 (10.4–22.2)
Blurred vision	11	7.7 (4.4–13.3)
Tremor	10	7.0 (3.8–12.4)
Lethargy	10	7.0 (3.8–12.4)
Headache	7	4.9 (2.4–9.8)
Nervousness	4	2.8 (1.1–7.0)
Loss of vision	4	2.8 (1.1–7.0)
Confusion	2	1.4 (0.4–5.0)
Cognitive impairment	2	1.4 (0.4–5.0)
**Vestibular-cerebellar**		
Dizziness	16	11.2 (7.0–17.4)
Dysarthria	4	2.8 (1.1–7.0)
Diplopia	3	2.1 (0.7–6.0)
Ataxia	3	2.1 (0.7–6.0)
**Respiratory**		
Bronchorrhea	8	5.6 (2.9–10.7)
**Digestive**		
Nausea	6	4.2 (1.9–8.9)
Vomiting	5	3.5 (0.2–7.9)
Gingival enlargement	3	2.1 (0.7–6.0)
Anorexy	2	1.4 (0.4–5.0)
Sialorrhea	1	0.7 (0.1–3.9)
**Dermatological**		
Acne	4	2.8 (1.1–7.0)
Rash	3	2.1 (0.7–6.0)
Hypertrichosis	1	0.7 (0.1–3.9)
**Endocrine/metabolic**		
Weight gain	3	2.1 (0.7–6.0)
Osteoporosis	1	0.7 (0.1–3.9)
**Hematological**		
Anemia	2	1.4 (0.4–5.0)
Leucopenia	1	0.7 (0.1–3.9)
**Genitourinary**		
Kidney stones	1	0.7 (0.1–3.9)
Increased urinary frequency	1	0.7 (0.1–3.9)
**Constitutional symptoms**		
Fatigue	3	2.1 (0.7–6.0)

ADR, adverse drug reaction; CI 95%, confidence interval 95%.

Regarding causality, 53.9% of the ADRs were classified as having a “possible” relationship, 45.5% were “probable” and only the 0.7% was considered “definite.”

Of the 143 ADRs, 68.5% were considered “preventable,” while the remaining 31.5% were classified as “non-preventable.”

Regarding the intensity of the evaluated ADRs, 46.9% were considered as “grade 1,” 44.1% were considered as “grade 2,” and the remaining 9.1% of ADRs were defined as “grade 3.”

Using the DoTS system, we found that 41.3% of the ADRs were “collateral,” 32.2% were classified as “hypersusceptibility” reactions and the remaining 26.6% were classified as “toxic effects.” However, it is important to mention that there was only objective evidence of supra-therapeutic plasma levels associated with phenytoin toxicity in a single patient (Calderon-Ospina et al., 2018).

According to the temporal relationship of ADRs established by the DoTS system, 86.7% of the ADRs were time-independent regarding the drug administration. The remaining 13.3% had a temporal relationship with drug administration as follows: 9.1% were “late” and 4.2% were “intermediate.” There were no documented “rapid,” “first dose,” “early,” or “delayed” ADRs.

Only in 5.6% of the ADRs it was not possible to identify a susceptibility factor predisposing the patient to ADRs. However, the remaining 94.4% of the ADRs had different susceptibility factors as follows: genetics (23.8%), age (43.4%), sex (53.2%), exogenous factors (55.2%), and disease (19.6%).

**Table 4** presents the classification of the ADRs found in the study.

**Table 4:** Classification of adverse reactions to phenytoin (n = 143).

Variable	n	%	IC 95%
**Causality**			
Definitive	1	0.7	0.1–3.9
Probable	65	45.5	37.5–53.6
Possible	77	53.9	45.7–61.8
**Preventability**			
Preventable	98	68.5	60.5–75-6
Non-preventable	45	31.5	24.4–39.5
**Intensity**			
Grade 1	67	46.9	60.5–75-6
Grade 2	63	44.1	36.2–52.3
Grade 3	13	9.1	5.4–14.9
**DoTS system (dose)**			
Hypersusceptibility	46	32.2	25.1–40.2
Collateral	59	41.3	33.5–49.5
Toxic effects	38	26.6	20.0–34.4
**DoTS system (time)**			
Not related	124	86.7	80.2–91.3
Intermediate	6	4.2	1.9–8.9
Late	13	9.1	5.4–14.9
**DoTS system (susceptibility)**			
Genetic	34	23.8	17.6–31.4
Age	62	43.4	35.5–51.6
Sex	76	53.2	45.0–61.1
Exogenous factors	79	55.2	47.1–63.2
Disease	28	19.6	13.9–26.8

CI 95%, confidence interval 95%; DoTS, dose, time, and susceptibility.

### Genotype—Phenotype Correlation

No significant differences were observed between the genetic variants *ABCB1-c.3435T>C* (rs1045642) and *SCN1A-IVS5-91G>A* (rs3812718) and the presence of DRE (non-response to antiepileptic treatment) (*p*=0.34) (**Table 5**). We also found no association between the genetic variants CYP2C9*2-c.430C>T and CYP2C9*3-c.1075A>C and ADRs (*p*=0.42) (**Table 6**).

**Table 5:** Genotype-phenotype correlation for the outcome of response to antiepileptic treatment with phenytoin.

	DRE (non-responding patients)	Responding patients
Patients homozygous for allele A of the *SCN1A* gene or patients homozygous for allele C of the *ABCB1* gene	16	13
Heterozygous and homozygous patients for the G allele of the *SCN1A* gene or heterozygous or homozygous patients for the T allele of the *ABCB1* gene	19	19

DRE, drug-resistant epilepsy; SCN1A, sodium voltage-gated channel alpha subunit 1; ABCB1, ATP binding cassette subfamily B member 1.

**Table 6:** Genotype-phenotype correlation for the outcome of adverse drug reactions to phenytoin.

	Patients with ADRs	Patients without ADRs
Intermediate/poor metabolizers for *CYP2C9* (homozygous or heterozygous for the allele CYP2C9*2 and the allele CYP2C9*3)	13	3
Normal metabolizers for *CYP2C9* (homozygous for the allele CYP2C9*1)	40	11

CYP2C9, cytochrome P450 family 2 subfamily C member 9; ADR, adverse drug reaction.

However, performing specific analyses of affected systems/organs using the one sample chi-square test with the Bonferroni correction, we found an association between decreased function alleles of *CYP2C9* and vestibular-cerebellar ADRs (dizziness, ataxia, diplopia, and dysarthria) (*p*=0.001). Regarding the other systems/organs affected by ADRs, we did not find a significant association: neurological ADRs (*p*=0.075), respiratory (*p*=0.186), digestive (*p*=0.017), dermatological (0.15), endocrine/metabolic (*p*=0.456), hematological (*p*=0.352), genitourinary (*p*=0.238), and constitutional symptoms (*p*=0.075).

## Discussion

This was an exploratory study with the aim to evaluate possible genetic determinants of response to anticonvulsant treatment with phenytoin in a group of Colombian patients with epilepsy. The three studied pharmacogenes provided valuable information on three critical points of the pharmacokinetic/pharmacodynamic pathways of this drug. Allelic and genotypic frequencies of *CYP2C9* were very similar to those found in another study carried out by our research group aimed at designing a pharmacogenetic algorithm, which involved 152 Colombian patients using warfarin (Galvez et al., 2018). However, the frequency of intermediate metabolizers in the present study was three times greater than the overall frequency of this phenotype reported in the literature (Caudle et al., 2014).

With respect to the *ABCB1-c.3435T>C* variant, results were similar to the overall frequency (Genome Reference Consortium, n.d). However, these results differ from those found by Velasco-Parra et al. in a case (n = 111) and control (n = 91) study on the relationship between this same variant and refractory epilepsy in a sample of 202 Colombian patients with epilepsy, in whom the frequency of the TT genotype was considerably lower (Velasco-Parra et al., n.d). It is possible that this can be explained by methodological differences such as the definition of DRE in both studies.

Finally, for the *SCN1A* gene, frequencies were similar to that reported in the literature (Genome Reference Consortium, n.d). As far as we know, no published studies have systematically evaluated the relationship between *SCN1A-IVS5-91G>A* polymorphism (rs3812718) and DRE in Colombian patients. However, the presence of heterozygosity for this polymorphism has been reported in a Colombian family affected by generalized epilepsy with febrile seizures (Pineda-Trujillo et al., 2005).

We did not find an association between *ABCB1-c.3435T>C* polymorphism and DRE. These results are consistent with different meta-analyses, which have yielded contradictory results (Bournissen et al., 2009; Haerian et al., 2010; Balan et al., 2014; Lv et al., 2014; Chouchi et al., 2017). This could be explained by the high degree of heterogeneity of the included studies, due to the varying definitions of DRE, the use of different AEDs, and the inclusion of different types and syndromes of epilepsy. Although some evidence from animal studies, clinical cases and genetic studies supports the hypothesis of overexpression of P-gp in the BBB as a cause of resistance to pharmacological treatment of epilepsy (Wang et al., 2016), the true implications of *ABCB1-c.3435T>C* polymorphism of the *ABCB1* gene have not yet been explained.

*ABCB1-c.3435T>C* polymorphism corresponds to a synonymous SNP in exon 26, which has been associated with an alteration in P-gp activity (Hoffmeyer et al., 2000; Drescher et al., 2002) and, when it appears in a haplotype, with reduced functionality (Salama et al., 2006). However, it has also been proposed that changes in phenotypic activity of P-gp expression can depend on the type and number of AEDs used, as well as the duration of treatment for each patient. This theory provide another biologically plausible explanation of the differences obtained in the studies (and meta-analyses) performed to evaluate the impact of *ABCB1*-*c.3435T>C* polymorphism on the therapeutic response to AEDs (Sisodiya and Goldstein, 2007).

Probably the most important result of this study was the association between the intermediate/poor metabolizer phenotype for *CYP2C9* and vestibular-cerebellar ADRs (dizziness, ataxia, diplopia, and dysarthria) (*p*=0.001). Due to the non-linear pharmacokinetics of phenytoin, consistent with a change in elimination kinetics from first to zero order (more evident in poor metabolizers) the role of the *CYP2C9* enzyme and its polymorphisms is critical for the generation of this type of ADRs (Silvado et al., 2018). Recently the FDA has recently adjusted the clinical pharmacology section of the phenytoin label to warn about the possibility of unusually high levels in patients with certain allelic variants in *CYP2C9* and *CYP2C19*(Research C for DE and. Science & Research (Drugs), n.d).

The pharmacovigilance results of this study coincide with previous reports (Chmielewska et al., 2013), with approximately 70% of ADRs being preventable, and the most frequent cause of adverse effects (and possibly non-response cases) being insufficient monitoring of plasma concentrations of phenytoin. This result is evident in the fact that according to DoTS evaluation almost three out of 10 ADRs were related to supra-therapeutic doses in our study. These figures highlight the necessary inclusion of non-genetic factors in pharmacogenetic studies.

As far as we know, this is the first clinical study with a pharmacogenomic and pharmacovigilance approach aimed at investigating the impact of three pharmacogenes and four distinct genetic polymorphisms in the therapeutic response to phenytoin, all of which were identified by an exhaustive literature review. This study used the ILAE criteria in order to have an objective, up-to-date and comprehensive definition of pharmacoresistance. Likewise, the ADRs were evaluated exhaustively with an active pharmacovigilance approach.

One of the limitations of the research project is that this was observational in nature, and plasma levels of phenytoin were only available in less than 20% of patients evaluated. The availability of this information for all patients would have allowed an accurate correlation between *CYP2C9* genotypes, plasmatic levels of phenytoin and therapeutic response, as well as a more precise attribution of causal relationship in ADRs according to the Naranjo algorithm, which includes supratherapeutic plasma levels of the suspect drug as one of the items to be evaluated. While phenytoin has a narrow therapeutic range, there is evidence to suggest that this drug should not be freely interchangeable (Gidal, 2009; Rediguieri and Zeredo, 2014). Therefore, the lack of control over which specific brand of phenytoin used by each patient may have introduced an additional bias into the study. Finally, the lack of statistical significance for many of the evaluated genotype-phenotype correlations, could be explained by the relatively small sample size or by the evaluation of a reduced number of polymorphisms within many hundreds of possible genetic variants. In addition, the analysis of the two other common *ABCB1* variants (c.1236C>T and c.2677G>T/A) in order to generate the corresponding haplotype would have been more relevant than the study of a unique *ABCB1* variant (Chouchi et al., 2017).

It has been hypothesized that synchronized neuronal hyperactivity (e.g., epileptic seizures) induces deep shifts in the epigenome and the expression of downstream genes. Epigenetic changes including DNA methylation, histone modifications, and microRNAs may be responsible for functional and structural changes within neural networks, as well as at the beginning of epilepsy and its progression to DRE (Kobow and Blümcke, 2018). In this way, the complexity of the problem to be studied (DRE) along with the limitations of the available data (e.g., lack of epigenetic data) may have limited the scope of this study.

Considering that the study was carried out in care centers specializing in the treatment of epilepsy could explain the higher frequency of patients with DRE, compared to what has been reported (Kwan et al., 2011; Balestrini and Sisodiya, 2018). Furthermore, the great majority of patients had epilepsy of unknown or structural/metabolic origin, and all of them received phenytoin to treat the condition, so the results obtained could not be extrapolated to other populations.

## Data Availability Statement

The raw data supporting the conclusions of this manuscript will be made available by the authors, without undue reservation, to any qualified researcher.

## Ethics Statement

The studies involving human participants were reviewed and approved by Comité de Ética en Investigación de la Universidad del Rosario. The patients/participants provided their written informed consent to participate in this study.

## Author Contributions

All authors have contributed significantly to the preparation of the manuscript, are in agreement with the content of the manuscript and agree to submission to the journal.

## Funding

This work was funded by Universidad del Rosario with grant number DVG-098.

## Conflict of Interest

PL declares that his present salary is paid by BIOPAS Laboratories for working in projects different to that described in the present manuscript.

The remaining authors declare that the research was conducted in the absence of any commercial or financial relationships that could be construed as a potential conflict of interest.

## Supplementary Material

Supplementary Data Sheet 1 Checklist for the evaluation of adverse reactions to phenytoin in the study patients.
