# Supplementary material for: Possible Genetic Determinants of Response to Phenytoin in a Group of Colombian Patients With Epilepsy
Source: Front Pharmacol. 2020 May 7;11:555. doi: 10.3389/fphar.2020.00555 (PMC7221122; doi:10.3389/fphar.2020.00555)
Supplement: Supplementary Data Sheet 1 — Checklist for the evaluation of adverse reactions to phenytoin in the study patients. [file DataSheet_1.pdf]

Checklist for the evaluation of ADRs

Patient code: _______ Date: _________

Mark with an "X" when you suspect any of the following adverse reactions to phenytoin:

**Neurological**	
Tremor	
Blurred vision	
Loss of vision	
Somnolence	
Lethargy	
Insomnia	
Dystonia	
Nervousness	
Agitation	
Dysarthria	
Headache	
Hypotonia	
Confusion	
Cognitive impairment	
**Vestibular-cerebellar**	
Dizziness	
Dysarthria	
Diplopia	
Ataxia	
**Psychiatric**	
Psychiatric and behavioral disorders	
**Respiratory**	
Bronchorrea	
**Cardiovascular**	
Palpitations (arrhythmia)	
**Digestive**	
Nausea	
Vomiting	
Anorexia	
Gingival enlargement	
Anorexy	
Increased appetite	
Sialorrhea	
Dysgeusia	
Impaired liver function tests. If so, specify test, result and date: ____________________________	
**Genitourinary**	
Kidney stones	
Increased urinary frequency	
**Hematological**	
Anemia<br>If so, specify result and date:<br>____________________________	
Leucopenia<br>If so, specify result and date:<br>____________________________	
Thrombocytopenia<br>If so, specify result and date:<br>____________________________	
Eosinophilia<br>If so, specify result and date:<br>____________________________	
Adenomegalies	
**Dermatological**	
Acne	
Alopecia	
Rash	
Exfoliative dermatitis	
Hypertrichosis	
Facial coarsening	
**Endocrine/metabolic**	
Weight gain	
Loss of weight	
Osteoporosis	
Syndrome of inappropriate antidiuretic hormone secretion	
Hyperglycemia<br>If so, specify result and date:<br>____________________________	
**Inmunological**	
Lupus-Like Syndrome	
**Constitutional symptoms**	
Fatigue	
**Other**<br>Specify: _________________	

Have you been at hospital since my last phone call? If so, explain why: ______________________________
